# Late Onset Telomere Biology Disorder Presenting With Pancytopenia, Immune Dysregulation, Interstitial Lung Disease and Alopecia

**DOI:** 10.1002/ajh.70234

**Published:** 2026-02-24

**Authors:** Bo A. Wan, Hamish Nicolson, Heather A. Leitch, Ryan J. Stubbins, Luke Y. C. Chen, Timothy Murray, Amy Trottier, Anthony Gador

**Affiliations:** ^1^ Division of Hematology University of British Columbia Vancouver British Columbia Canada; ^2^ Division of Hematopathology University of British Columbia Vancouver British Columbia Canada; ^3^ Coastal Program for Rare Inflammatory Diseases Dalhousie University Halifax Nova Scotia Canada; ^4^ Division of Hematology and Hematologic Oncology, Department of Medicine Dalhousie University Halifax Nova Scotia Canada; ^5^ Department of Radiology University of British Columbia Vancouver British Columbia Canada; ^6^ Providence Undiagnosed and Rare Conditions Clinic, St. Paul's Hospital and the Division of General Internal Medicine University of British Columbia Vancouver British Columbia Canada

**Keywords:** alopecia, lymphohistiocytosis, pancytopenia, pulmonary fibrosis, telomere biology disorders

## 1 Case Presentation

A 60‐year‐old Eastern European woman presented to primary care with progressive fatigue and dyspnea. Her family history includes no known hematologic concerns; mother: died age 39 of an accident; father: alive aged 91; sister: alive, aged 63; her two daughters aged 39 and 33 are healthy. Physical examination showed a slim female of average height with moderate, diffuse alopecia. Bloodwork revealed pancytopenia, progressive since detection 2 years previously: hemoglobin 85 g/L [reference 115–150], MCV 103 fL [82–98], platelets 69 × 10^9^/L [150–400], neutrophils 1.2 × 10^9^/L [2–7]. Her CBC was normal greater than 2 years prior.

**FIGURE 1:**
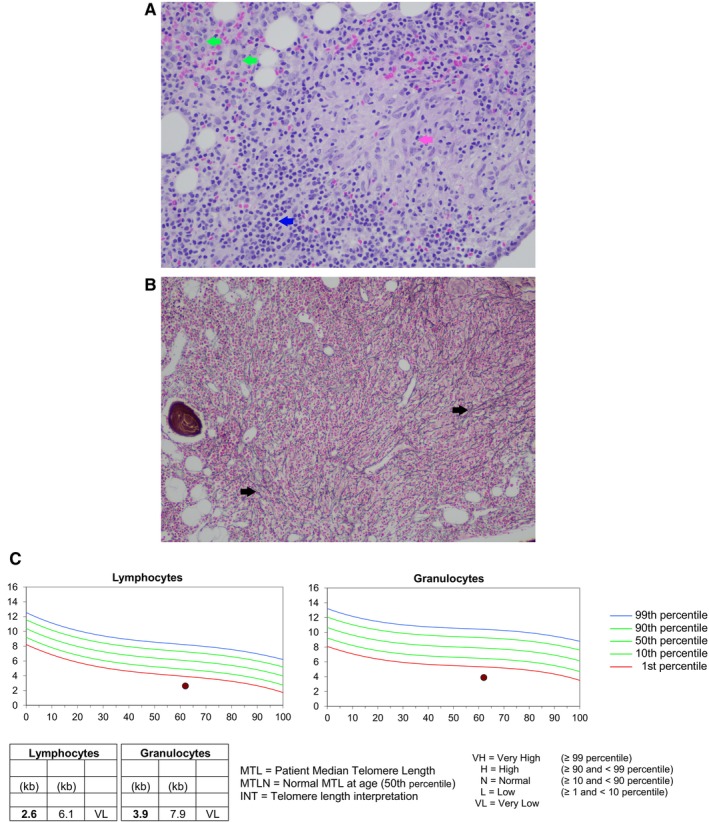
Bone marrow biopsy showing (A) a cluster of epithelioid histiocytes (granuloma; pink arrow) surrounded by small lymphocytes (blue arrow) and plasma cells (green arrows) (Hematoxylin and eosin ×200) and (B) hypercellular bone marrow showing a diffuse meshwork of reticulin fibrosis (black arrow) against a background of lymphohistiocytic infiltration (Gomori's silver ×100). Telomere length testing by Flow‐FISH (C) demonstrated median telomere lengths of 2.6 kb (< 1st percentile, normal median telomere length for age (MTLN) 6.1 kb) in the lymphoid series and 3.3 kb (< 1st percentile, MTLN 7.9 kb) in the myeloid series, consistent with a telomere biology disorder.

**FIGURE A1:**
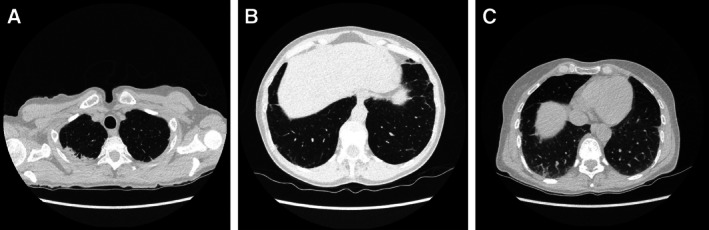
CT chest. Axial non‐contrast inspiratory CT chest at the lung apex (A) demonstrates apical subpleural consolidation and mild fibrosis, bilateral with a right‐sided predominance. Axial image from the lung base of the same study (B) demonstrates minimal basal subpleural and bronchocentric reticular changes, without honeycombing or ground‐glass change. On dedicated expiratory view, no significant air‐trapping (C).

**FIGURE A2:**
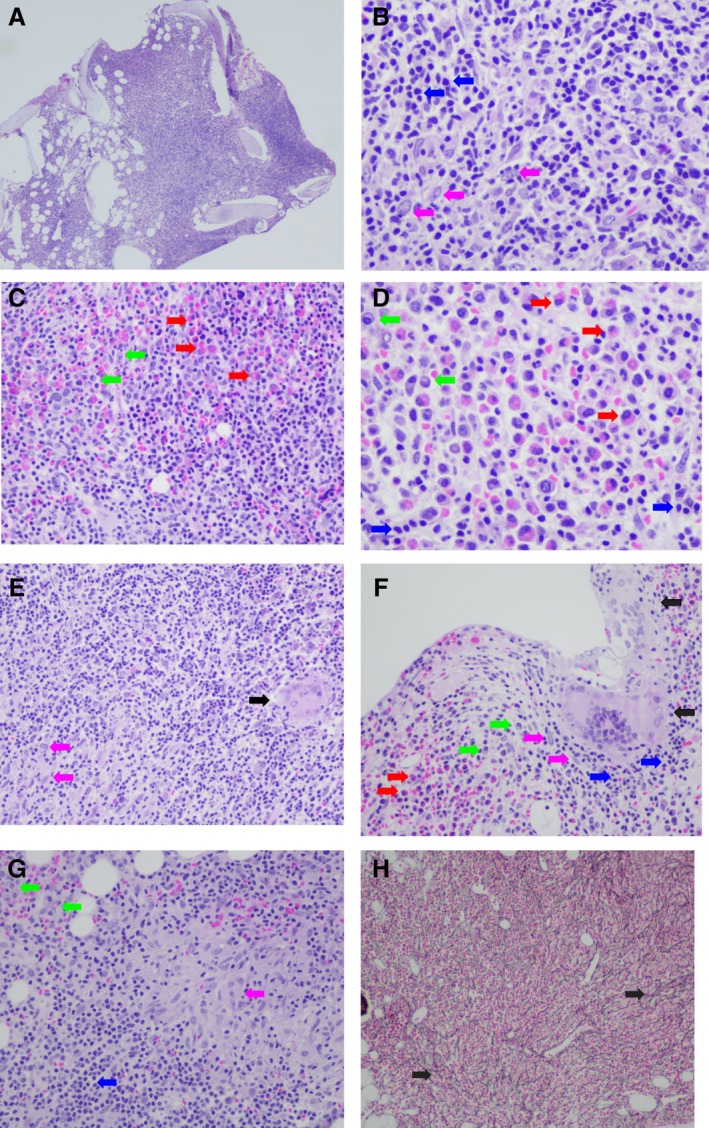
Additional bone marrow images. (A) Trephine bone marrow biopsy showing hypercellularity. The normal marrow architecture is effaced by a lymphohistiocytic infiltrate (Hematoxylin and eosin ×40). (B) Lymphohistiocytic infiltrate comprised of an admixture of small mature lymphocytes (blue arrow) with dark condensed nuclei and epithelioid histiocytes (pink nuclei) with ovoid nuclei and small visible nucleoli (Hematoxylin and eosin ×400). (C) Focal area of the bone marrow showing increased eosinophils (red arrow) and plasma cells (green arrow) interfacing with the lymphohistiocytic infiltrate (Hematoxylin and eosin ×200). (D) Higher power magnification showing an admixture of eosinophils (red arrow), plasma cells (green arrow), and small lymphocytes (blue arrow) (Hematoxylin and eosin ×400). (E) Lymphohistiocytic infiltrate with epithelioid histiocytes (pink arrow) and a multinucleated giant cell (black arrow) (Hematoxylin and eosin ×200). (F) Focal area of increased eosinophils (red arrow) and plasma cells (green arrow) adjacent to large multinucleated giant cells (black arrow) surrounded by small lymphocytes (blue arrow) and epithelioid histiocytes (pink arrow) (Hematoxylin and eosin ×200). (G) Cluster of epithelioid histiocytes (granuloma; pink arrow) surrounded by small lymphocytes (blue arrow) and plasma cells (green arrow) (Hematoxylin and eosin ×200). (H) Hypercellular bone marrow showing a diffuse meshwork of reticulin fibrosis (black arrow) against a background of lymphohistiocytic infiltration (Gomori's silver ×100).

Extensive hematologic, autoimmune, and infectious work‐up revealed polyclonal hypergammaglobulinemia and a detectable low‐level EBV viremia of < 1000 copies/mL (Appendix A: Figures A1 and A2). Two bone marrow biopsies done a year apart both showed hypercellularity at nearly 100% with reticulin fibrosis (Figure 1A,B). There was near complete effacement of normal marrow architecture by an atypical infiltrate composed of abundant small mature lymphocytes and scattered histiocytes. T‐cell clonality was positive. The significance of the lymphohistiocytic infiltrate was unclear but not felt to be consistent with a lymphoma. A third bone marrow biopsy done 2 years after the index biopsy demonstrated a mildly hypocellular marrow (30%–40% cellularity) without dysplasia, suggesting a patchy involvement with this lymphohistiocytic infiltrate. Karyotype was normal, and a next‐generation sequencing panel demonstrated no somatic variants. She underwent pan‐CT, PET scan, bone scan, and upper GI endoscopy; together these showed interstitial lung disease (ILD) with mild apical subpleural scarring and fibrosis.

After evaluation with hematology, respirology, and infectious diseases without a diagnosis, she was seen by the rare diseases clinic in our center.

She underwent gene panel sequencing (Blueprint immune and cytopenia panel) which revealed a heterozygous variant in *RTEL1* NM_032957 transcript (c.805del, pp.Thr269Glnfs*3) with a VAF of 47%. This variant is absent in gnomAD and causes a frameshift in exon 9 (of 35 total exons), resulting in a premature stop codon and is predicted to lead to loss of normal protein function. This was curated as likely pathogenic by the reporting laboratory, meeting criteria for PVS1 and PM2_supp.

*RTEL1* encodes a DNA helicase involved in DNA repair and telomere maintenance; *RTEL1* variants are associated with telomere biology disorders (TBDs). TBDs resulting from deleterious germline *RTEL1* variants can exhibit autosomal dominant (AD) or autosomal recessive (AR) modes of inheritance. Patients with AR *RTEL1*‐associated TBDs present with a more severe phenotype, often associated with the classic mucocutaneous triad or Hoyeraal–Hreidarsson syndrome. Patients with AD *RTEL1*‐associated TBDs can have a more subtle presentation and can manifest later in life.

This patient's presentation was consistent with an autosomal dominant TBD. Telomere length testing by Flow‐FISH (Repeat Diagnostics) demonstrated median telomere lengths of 2.6 kb (< 1st percentile, normal median telomere length for age (MTLN) 6.1 kb) in the lymphoid series and 3.3 kb (< 1st percentile, MTLN 7.9 kb) in the myeloid series (Figure 1C). The *RTEL1* variant was identified in the lymphocyte fraction on sorted lymphocyte testing as well as on cultured fibroblasts, confirming its germline origin. She is currently on observation and a donor search is underway for potential allogeneic stem cell transplant in the future should she develop consequences from immune dysregulation, persistent infections, refractory cytopenias despite conservative management, or clonal myeloid findings.

This case highlights that germline variants and hereditary marrow failure disorders may manifest clinically in older adulthood. The identification of her polyclonal hypergammaglobulinemia and lymphohistiocytic infiltrate may be a potential feature of TBDs, potentially related to immune dysregulation. With increasing accessibility, genetic analysis should be considered in the investigation of unusual clinical presentations that have escaped explanation with conventional work‐up.

## Author Contributions

N.H., H.A.L., R.J.S., L.Y.C.C., A.G. were involved in patient care. B.A.W. and L.Y.C.C. drafted the manuscript. H.N. provided the hematopathology figures and pathology expertise. T.M. provided the radiology figures and expertise. A.T. provided inherited bone marrow disorders expertise. All authors edited and approved the final manuscript. The patient provided written consent for publication of the case.

## Ethics Statement

The authors have written informed consent from the patient to submit this clinical picture for publication. Ethics approval was not required by our institutional ethics review board for case reports.

## Conflicts of Interest

The authors declare no conflicts of interest.

## Appendix A Results of Additional Investigations

Peripheral blood flow cytometry showed an expanded NK‐cell population and a positive T‐cell clonality study. The significance of the lymphohistiocytic infiltrate was unclear but was not felt to be consistent with a lymphoma.

There was a mild reduction in Diffusing capacity for carbon monoxide (DLCO 71% predicted), spinal compression fractures with osteoporosis were seen on whole body CT, and PET scan showed low‐grade increased FDG‐uptake in the spleen and throughout the marrow, overall negative for malignancy. The Blueprint genetic immunodeficiency and cytopenia panel identified a pathogenic heterozygous RTEL1 truncating deletion at c805, p.Thr269Glnfs*3 of exon 9. Additional findings include a pathogenic heterozygous ERCC2 c1703_1704 deletion and variants of uncertain significance (JAK2, NOD2, CFTR).

**Table:**

Additional bloodwork	Result
SPEP	Slight polyclonal increase in gamma globulins. No paraprotein band is seen
C3	0.97 g/L [1–1.9]
C4	0.20 g/L [0.09–0.5]
Free Kappa	54.0 mg/L [3.3–19.4]
Free Lambda	28.9 mg/L [5.7–26.3]
Kappa/lambda ratio	1.87
IgG	25.4 g/L [7–16]
IgA	4.43 g/L [0.7–4]
IgM	1.23 g/L [0.4–2.30]
IgE	22 μg/L [< 430]
IgG1 subclass	14.6 g/L [2.8–8]
IgG2 subclass	7.59 g/L [1.15 = 5.70]
IgG3 subclass	1.49 g/L [0.24–1.25]
IgG4 subclass	0.815 g/L [0.052–1.250]
Tryptase	7.91 μg/L [< 11.1]
Haptoglobin	1.02 g/L [0.3–2]
Ferritin	93 μg/L [15–247]
Iron saturation	0.22
Vitamin B12	441 pmol/L [153–655]
Hemoglobin electrophoresis	There is no evidence of thalassemia or hemoglobinopathy
Alpha 1 antitrypsin	1.70 g/L [0.9–2.0]
Beta‐2‐microglobulin	2 mg/L [< 2.2]

Infectious work‐up	Result
Coccidioides IgG, IgM	Nonreactive
Histoplasma	Nonreactive
Brucella	Nonreactive
*Helicobacter pylori* Stool Ag	Nonreactive
Syphilis EIA	Nonreactive
HSV1	Past infection
HSV2	Negative
HTLV1+ HTLVII	Nonreactive
TB QuantiFERON	Nonreactive
HIV	Nonreactive
Hepatitis B	No evidence of HBV infection, patient is immune to HBV likely from vaccination
Hepatitis C	Nonreactive
Parvovirus	Negative
EBV PCR	< 1000 copies/mL
EBV Capsid IgG	Reactive
VZV	Immune to VZV
*Treponema pallidum*	Nonreactive
CMV IgG	Reactive
CMV PCR	< 35
Filaria	Nonreactive
Leishmania	Nonreactive
Schistosoma	Nonreactive
Toxoplasma	Nonreactive
Strongyloides	Nonreactive
Parvovirus B19	Evidence of past infection

Autoimmune	Result
PR3	< 0.2 AI [< 1]
MPO	< 0.2 AI [< 1]
ANA titre	Neg
dsDNA	1 IU [< 4]
CCP	< 0.5 U/mL [< 3]
TTG	< 0.5 U/mL [< 12]
RF	< 19 kU/L [< 30]

Heavy metal screening	Result
Ceruloplasmin	350 mg/L [200–500]
Lead	0.04 μmol/L [< 0.48]
Mercury	< 5 nmol/L [< 29]
Zinc	9.5 μmol/L [9.2–15.4]
Arsenic creatinine urine ratio	37 μmol/mol/Cr [< 92]

